# Randomised trial evaluating chemotherapy alone or chemotherapy and a novel monoclonal antibody for canine T‐cell lymphoma: A multicentre US study

**DOI:** 10.1002/vro2.49

**Published:** 2022-11-01

**Authors:** Margaret L. Musser, Craig A. Clifford, Philip J. Bergman, Laura S. Treml, Lydia C. Cook McAnulty, Elizabeth A. McNiel, Chad M. Johannes

**Affiliations:** ^1^ Iowa State University College of Veterinary Medicine Lloyd Veterinary Medical Center Ames Iowa USA; ^2^ Blue Pearl Malvern/Hope Veterinary Specialists Malvern Pennsylvania USA; ^3^ Department of Clinical Studies VCA, Katonah Bedford Veterinary Center Bedford Hills New York USA; ^4^ Knoell Animal Health LLC Kansas Missouri USA; ^5^ Dechra Pharmaceuticals Fort Worth Texas USA; ^6^ VCA Advanced Veterinary Care Center Fishers Indiana USA; ^7^ Colorado State University College of Veterinary Medicine and Biomedical Sciences, Flint Animal Cancer Center Fort Collins CO USA

## Abstract

**Background:**

Canine peripheral nodal T‐cell lymphoma is considered chemotherapy resistant and carries a relatively poor prognosis. Prospective evaluations reporting the impact of chemotherapy on progression‐free survival (PFS) and overall survival time for dogs with T‐cell lymphoma are lacking. This study examined the impact of L‐CHOP (L‐asparaginase, doxorubicin, cyclophosphamide, vincristine, prednisone) chemotherapy or L‐CHOP in combination with AT‐005, a US Department of Agriculture‐licensed caninised monoclonal antibody, on PFS and response rates in dogs with clinical intermediate‐ and high‐grade peripheral nodal T‐cell lymphoma.

**Methods:**

A prospective, randomised, placebo‐controlled, investigator‐ and owner‐blinded, multicentre study was completed. All dogs received a 19‐week L‐CHOP chemotherapy protocol with randomisation (1:1) into placebo or AT‐005 groups. Response was evaluated via the Veterinary Cooperative Oncology Group criteria for canine lymphoma.

**Results:**

Forty‐nine dogs were enrolled (25 received placebo and 24 received AT‐005). Most demographic factors were similar between the two groups, with the exception that more dogs with stage IV and V disease were treated with AT‐005 (34% vs. 8%; *p* = 0.03). Median PFS was 103 days (95% confidence interval [CI], 56–118) in the placebo group versus 64 days (95% CI, 36–118) in the AT‐005 group. The overall response rate (ORR) for all dogs was 98% (48 of 49); complete response rate in the placebo group (64%) was not different from the AT‐005 group (67%).

**Conclusions:**

To the best of the authors’ knowledge, this is the first prospective study to document that treatment with L‐CHOP chemotherapy, with or without AT‐005, may result in a high ORR, but relatively brief PFS in dogs with clinical intermediate‐ and high‐grade T‐cell lymphoma.

## INTRODUCTION

Canine peripheral nodal T‐cell lymphoma (LSA) is an aggressive cancer that appears to be resistant to common treatment protocols, such as L‐CHOP (L‐asparaginase, doxorubicin, cyclophosphamide, vincristine, prednisone).^1^ Despite multiagent chemotherapy, dogs with T‐cell LSA have a poorer prognosis compared to those with B‐cell LSA, typically having both shorter remission and overall survival times.^1^ In general, B‐cell LSA dogs treated with CHOP chemotherapy have a median survival time of 300–365 days,^2^ while dogs with T‐cell LSA have median survival times of 235 days or less.^3^ These statistics have typically been derived from retrospective studies with confounding variables (inclusion of B‐cell dogs, use of CHOP only without L‐asparaginase, comparison of L‐CHOP to alternative treatment options), limiting the number of evaluable T‐cell dogs treated with L‐CHOP alone.^1^, ^3^, ^4^, ^5^ Thus, prospective studies evaluating naïve T‐cell LSA dogs with various chemotherapy protocols are required to truly understand the impact, or lack thereof, of chemotherapy for peripheral nodal T‐cell LSA cases.

This apparent resistance and lack of response durability to chemotherapy is attributed to decreased efficacy of certain CHOP chemotherapy drugs, including cyclophosphamide,^6^, ^7^ doxorubicin^8^ and prednisone.^9^ In addition, dogs with T‐cell LSA express higher levels of adenosine triphosphate‐binding cassette (ABC) transporter proteins, which are associated with vincristine, doxorubicin and prednisone resistance,^10^ potentially explaining the decreased response and survival times in dogs with T‐cell LSA treated with L‐CHOP. Thus, treatment protocols that include drugs not actively effluxed by the ABC transporter proteins have been investigated to try to overcome this natural resistance in dogs with T‐cell LSA. Protocols including lomustine or lomustine, vincristine, procarbazine, prednisone (LOPP) are potentially promising, with several studies resulting in numerical survival advantages compared to L‐CHOP alone.^5^, ^11^, ^12^, ^13^ Unfortunately, mechlorethamine, vincristine, procarbazine, prednisone (MOPP) protocols have not consistently conferred a survival advantage or improved remission rates.^4^, ^14^ Similarly, novel agents and approaches including rabacfosadine,^15^, ^16^ verdinexor^17^ and autologous bone marrow transplants^18^ have failed to significantly improve overall survival times. Thus, in addition to confirmatory prospective information about the impact of currently used chemotherapy protocols, innovative treatment approaches are necessary to improve outcome in dogs with T‐cell LSA.

Human peripheral T‐cell LSA is rare, aggressive and biologically similar to canine clinical high‐grade T‐cell LSA. It is a heterogenous group of diseases that are further subdivided into approximately 20 categories based on specific staining characteristics or anatomical distribution.^1^ Treatments vary, they typically start with regimens based on multiagent chemotherapy protocols, including CHOP.^19^ Interestingly, CHOP confers a remission rate of only 50%–65% in people,^20^ suggesting humans and dogs with T‐cell lymphoma share inherently poor responses to anthracycline‐based protocols. In humans, alternative treatment options investigated include autologous bone marrow transplants, novel chemotherapeutics (pralatrexate), histone deacetylase inhibitors (romidepsin and belinostat)^19^ and chimeric antigen receptor T‐cell therapy.^21^ The monoclonal antibodies alemtuzumab (anti‐CD52)^22^ and brentuximab vedotin (anti‐CD30) have also shown objective responses in relapsed T‐cell LSA.^23^

Due to the success of the monoclonal antibody (mAb) rituximab (anti‐CD20) in human B‐cell LSA and the apparent response to brentuximab vedotin in relapsed human T‐cell LSA, mAb development is being explored in veterinary oncology. The canine T‐cell (anti‐CD52) lymphoma mAb, AT‐005 (Tactress; Aratana Therapeutics, Kansas City, KS, USA), received conditional (2014) and full (2016) licensure^24^ by the US Department of Agriculture (USDA) for the treatment of dogs with T‐cell lymphoma.

For the study described here, dogs with T‐cell LSA were prospectively recruited to evaluate the progression‐free survival (PFS) following administration of either L‐CHOP or L‐CHOP with AT‐005. The hypothesis was the addition of AT‐005 would result in a longer PFS when compared to L‐CHOP alone.

## MATERIALS AND METHODS

### Enrollment criteria and treatment stratification

Dogs with cytologically diagnosed stage II or higher peripheral nodal T‐cell LSA were prospectively enrolled in a randomised, placebo‐controlled, investigator‐ and owner‐blinded, multicentre clinical trial. Within this publication, peripheral nodal T‐cell LSA refers to the clinical definition of peripheral T‐cell LSA and not the histological definition. All dogs received a 19‐week L‐CHOP chemotherapy protocol and were randomised to receive either AT‐005 or a placebo contemporaneously, starting at week 2 of the L‐CHOP protocol (Table 1). Enrolled dogs were followed for 18 months or until disease progression or treatment intolerance occurred.

**TABLE 1:** Treatment protocol

Week	Drug	Dose
Screening/1	L‐asparaginase Vincristine Prednisone	10,000 IU/m^2^ IM (maximum of 10,000 IU per dog) 0.5 mg/m^2^ IV 2.0 mg/kg PO daily, not to exceed 60 mg total
2	Cyclophosphamide Prednisone Placebo/AT‐005	250 mg/m^2^ PO 1.5 mg/kg PO daily Based on weight, twice weekly, IV
3	Vincristine Prednisone Placebo/AT‐005	0.7 mg/m^2^ IV (if well‐tolerated previously) 1 mg/kg PO daily Based on weight, twice weekly, IV
4	Doxorubicin Prednisone Placebo/AT‐005	Dogs ≥10 kg: 30 mg/m^2^ IV Dogs ≤10 kg: 25 mg/m^2^ IV 0.5 mg/kg PO daily Based on weight, twice weekly, IV
5	Placebo/AT‐005	Based on weight, twice weekly, IV
6	Vincristine	0.7 mg/m^2^ IV
7	Cyclophosphamide Placebo/AT‐005	250 mg/m^2^ PO Based on weight, once, IV
8	Vincristine	0.7 mg/m^2^ IV
9	Doxorubicin Placebo/AT‐005	Dogs ≥10 kg: 30 mg/m^2^ IV Dogs ≤10 kg: 25 mg/m^2^ IV Based on weight, once, IV
11	Vincristine Placebo/AT‐005	0.7 mg/m^2^ IV Based on weight, once, IV
12	Cyclophosphamide	250 mg/m^2^ PO
13	Vincristine Placebo/AT‐005	0.7 mg/m^2^ IV Based on weight, once, IV
14	Doxorubicin	Dogs ≥10 kg: 30 mg/m^2^ IV Dogs ≤10 kg: 25 mg/m^2^ IV
16	Vincristine	0.7 mg/m^2^ IV
17	Cyclophosphamide	250 mg/m^2^ PO
18	Vincristine	0.7 mg/m^2^ IV
19	Doxorubicin	Dogs ≥10 kg: 30 mg/m^2^ IV Dogs ≤10 kg: 25 mg/m^2^ IV

Abbreviations: IM, intramuscularly; IV, intravenously; PO, orally.

Dogs of any breed or sex, over 1 year of age and weight of 2 kg or more, with naïve, clinical intermediate‐ or high‐grade, measurable peripheral nodal T‐cell LSA (stage II or higher) were eligible for screening. Screening included lymph node flow cytometry (completed at Colorado State University, Fort Collins, CO, USA) and/or Tru‐Cut lymph node histopathology with immunohistochemistry (completed through VDx Veterinary Diagnostics and Preclinical Research Services, Davis, CA, USA), a complete blood count (CBC), chemistry panel (CHEM) and a urinalysis (UA). All available LSA lymph node biopsies were evaluated on haematoxylin and eosin‐stained sections and graded using previously reported criteria.^25^ Dogs were required to have at least one peripherally located lymph node measuring 2 cm or more in longest diameter for evaluation. A previously published modified Eastern Comparative Oncology Group (ECOG) constitutional performance score was used to screen potential cases.^26^ A score of 0 (normal activity), 1 (restricted activity; decreased activity from pre‐disease status) or 2 (compromised, ambulatory for vital activities) was confirmed prior to enrollment. Cases were excluded if they had an ECOG performance score of 3 or 4; received glucocorticoid therapy for more than 7 days prior to enrollment, prior chemotherapy, immunotherapy or molecularly targeted therapy; had any uncontrolled medical condition that would be disruptive to the intent and objectives of the study; were pregnant or likely to become pregnant; were participating in alternative studies; or if the dog was unavailable for the entire study duration. Dogs were allowed to be withdrawn from the study if one or more of the following occurred: an adverse event (AE); lack of expected efficacy; treatment failure; withdrawal of owner consent; or other justifiable reasons. Once clinical intermediate to high‐grade T‐cell LSA and eligibility were confirmed, thoracic radiographs were completed. Additional staging, including abdominal imaging and bone marrow analysis, was at the discretion of the attending clinician.

Dogs were randomised to the placebo or AT‐005 groups according to a blocked 1:1 randomisation design on order of enrollment among all sites. A random treatment allocation plan was generated using Microsoft Excel (Redmond, WA, USA). The investigator at each study site and the dog owner were blinded and had no knowledge of treatment group assignments. The site coordinator and at least one other member of the study team at each site were not blinded to act as test article dispensers, with the responsibility of correctly dosing all study dogs and preparing either placebo or AT‐005 for administration. Documentation was made in the medical record that the dog was participating in a clinical trial; no indication of treatment assignment was given. All personnel evaluating clinical or laboratory responses to therapy were blinded regarding the treatment given.

### AT‐005 chimeric monoclonal antibody design

The canine chimeric mAb was developed by Vet Therapeutics (acquired by Aratana Therapeutics, now Elanco Animal Health; the antibody is not currently available to veterinarians on the open market).^27^ Briefly, the canine CD52 coding sequence was cloned following isolation from canine peripheral blood mononuclear cells. Total RNA was extracted and cDNA was synthesised. The coding region was then amplified by PCR. Antibodies to CD52 were raised using polypeptides encompassing CD52 amino acid sequences. Chinese hamster cells, human embryonal kidney cells and mouse embryonal fibroblasts were transfected with an expression vector encoding CD52; anti‐CD52 monoclonal antibodies were generated by immunisation of mice to raise immunoglobulins specific for canine CD52. Monoclonal antibody‐producing hybridomas were expanded and purified using one clone following standard techniques.^28^ The antibody and evidence of binding, was provided by the inventor of the antibody to Aratana Therapeutics.

### Placebo or AT‐005 administration

Placebo (0.9% NaCl solution) or AT‐005 (5 mg/ml) was administered based on weight as a 15–30 min infusion. AT‐005 was provided as an injectable, ready to use sterile liquid. Dogs weighing between 2 and 15 kg received 7.5 ml (37.5 mg; 1 vial equivalent) placebo/AT‐005. Those between 15.1 and 30 kg received 15 ml (75 mg) placebo/AT‐005; between 30.1 and 45 kg received 22.5 ml (112.5 mg) placebo/AT‐005; those between 45.1 and 60 kg received 30 ml (150 mg) placebo/AT‐005. The administration schedule is outlined in Table 1. Pretreatment with diphenhydramine (2 mg/kg subcutaneously 30 min prior to infusion) was administered if the dog was not receiving prednisone. It was also recommended to monitor each case for 30 min after treatment to evaluate for allergic reactions.

Chemotherapy dose adjustments were allowed if they were determined to be medically necessary by the site investigator. Dose reductions of more than 25% or delays longer than 7 days were permitted if approved by the study medical director. L‐CHOP chemotherapy resumed as scheduled following the delay. Despite any chemotherapy delay, no delays in placebo or AT‐005 were allowed unless an AE was considered to be related to the study treatment.

During the study, dogs did not receive concomitant drugs targeted at the treatment of cancer including any holistic therapy, immunotherapy, radiation therapy, small molecule inhibitors or other investigational therapies unless approved by the medical director and sponsor of the clinical trial. Antiemetics, antidiarrhoeals and antibiotics were permitted as preemptive or symptomatic clinical management.

Adverse events were graded according to the Veterinary Cooperative Oncology Group (VCOG) common terminology criteria for AEs following chemotherapy or biological antineoplastic therapy in dogs and cats (version 1.1).^29^ For the purposes of this study, a serious AE was defined as one that resulted in hospitalisation longer than 24 h or significant disability or morbidity, was life threatening or resulted in death.

A minimum of one and a maximum of five target lymph nodes were measured at each study visit for evaluation of efficacy. The longest diameter was measured and the mean sum was calculated. Progression‐free survival was the primary efficacy endpoint and was calculated from the time of first dose of chemotherapy until the time of progressive disease (PD). The VCOG response evaluation criteria for peripheral nodal lymphoma in dogs was used to define objective response as complete response (CR), partial response (PR), PD or stable disease (SD).^30^ Overall response rate (ORR) was defined as those dogs achieving a PR or CR. Recheck evaluations included a physical examination with lymph node measurements and CBC/CHEM/UA every other month until disease progression was confirmed.

### Statistical analysis

This prospective study was powered (90%) to detect a 50% increase in PFS in dogs receiving AT‐005 versus placebo. Kaplan–Meier methodology was used to generate survival curves for the overall population based on treatment protocol, stage (stage III or less, greater than stage III), histological grade (intermediate, high), breed (boxer, all other) and geographical region (excluding Southern California and the Rocky Mountain region as there were less than two dogs in at least one of the treatment arms). As one of the goals of this study was to report on the overall survival of dogs with clinical high‐grade T‐cell LSA treated with L‐CHOP, it was elected to group stages II–III together based on previous studies that reported a significant difference between stage III clinical high‐grade B‐cell LSA and stage III clinical high‐grade T‐cell LSA.^31^ Cases were censored if they were alive at data evaluation. Log rank test for two datasets with two‐tailed *p*‐values was used to compare survival curves. A *p*‐value of less than 0.05 was considered significant (LIFETEST, SAS, SAS Institute, Cary, NC, USA; version 9.4).

Group comparisons for other variables were performed and compared with Fisher's exact or chi‐square tests for categorical variables, Cochran–Mantel–Haenszel tests for ordinal variables and Mann–Whitney *U*‐tests for continuous variables. Wilcoxon matched‐pair signed rank tests were used for changes from baseline for each treatment group, separately, for continuous variables. Fisher's exact test was used to evaluate the influence of treatment on response rates (CR at any point). Duration of response calculated as the number of days from the first CR or PR to PD was also evaluated. A one‐way ANOVA was used to evaluate the influence of treatment on duration of response. Variables evaluated for impact on response rate and duration included AT‐005 versus placebo, stage (stage III or less, greater than stage III), histological grade (intermediate, high), breed (boxer, all other) and geographical region (excluding Southern California and the Rocky Mountain region as there were less than two dogs in at least one of the treatment arms).

## RESULTS

**FIGURE 1:**
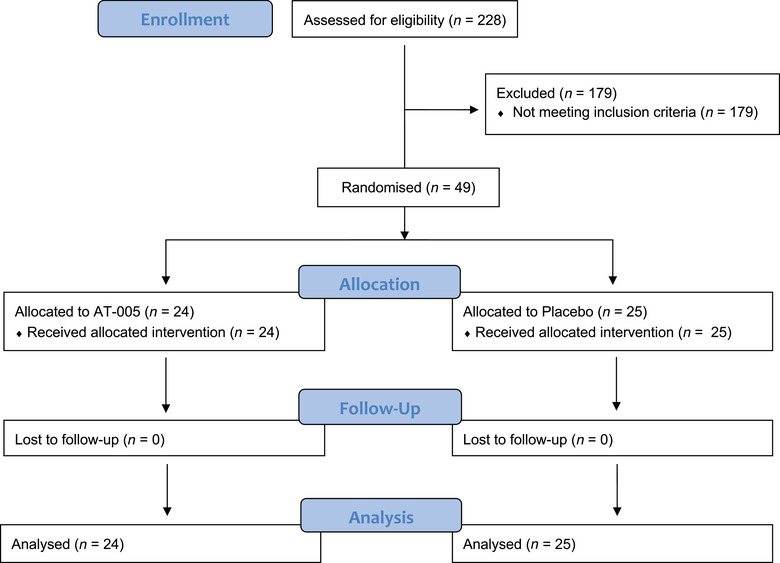
Enrollment flow diagram

Two hundred and twenty‐eight dogs at 11 study sites were screened for enrollment between June 2014 and March 2016. Forty‐nine dogs with confirmed T‐cell LSA were enrolled. The most common cause for screen failure was a phenotypical finding of B‐cell lymphoma based on flow cytometry. Twenty‐five were randomised to receive placebo and 24 to the AT‐005 group (Figure 1). Thirty‐four males and 15 females were enrolled. Median weight was 34 kg (range: 5–65) while median age was 7 years (range: 3–12). Eleven dogs had histologically confirmed intermediate‐grade LSA, 10 had intermediate to high‐grade and 21 had high‐grade LSA. For seven dogs, tissue was not available and thus grade was not histologically determined. In these demographics, there were no differences between the two treatment groups. Overall, five dogs had stage II disease, 34 stage III, seven stage IV and three stage V. In the AT‐005 group, there were significantly more stage IV and V LSA dogs compared to placebo (34% vs. 8%; *p* = 0.03). Twenty‐one dogs had a performance score of 0 at enrollment, while 27 had a score of 1 and one dog had a score of 2. A larger number of dogs with a performance score of 1 or 2 were treated with AT‐005 versus placebo (67% vs. 48%; *p* = 0.18) (Table 2). Concomitant treatments were varied and most commonly included medications used to control clinical side effects associated with L‐CHOP chemotherapy (antiemetic and antidiarrhoeal medications).

**TABLE 2:** Demographic summary of cases enrolled

		By treatment group	
	All (*n* = 49)	AT‐005 (*n* = 24)	Placebo (*n* = 25)
Weight (kg), median (range)	34 (5–65)	32 (13–43)	35 (6–65)
Age (years), median (range)	7 (3–12)	7 (4–12)	7 (3–11)
Sex			
Male	34 (69%)	17 (71%)	17 (68%)
Female	15 (31%)	7 (29%)	8 (32%)
**Lymphoma stage**			
II	5 (10%)	2 (8%)	3 (12%)
III	34 (70%)	14 (58%)	20 (80%)
IV	7 (14%)	5 (21%)	2 (8%)
V	3 (6%)	3 (13%)	0 (0%)
**Performance score at enrollment**			
0	21 (43%)	8 (33%)	13 (52%)
1	27 (55%)	16 (67%)	11 (44%)
2	1 (2%)	0 (0%)	1 (4%)
**Histological grade**	(*n* = 42)	(*n* = 20)	(*n* = 22)
Intermediate	11 (26%)	3 (15%)	8 (36%)
Intermediate to high	10 (24%)	5 (25%)	5 (23%)
High	21 (50%)	12 (60%)	9 (41%)

The median PFS time in the placebo group was 103 days versus 64 days in the AT‐005 group (*p* = 0.8163). There were no significant differences in PFS with any of the variables evaluated (Table 3). The ORR for the entire population was 98%, so no statistical analysis comparing ORR between the placebo group and the AT‐005 group was completed. Instead, variables potentially impacting the rates of CR between the groups were evaluated. No factors influenced the CR (Table 4) or duration of response.

**TABLE 3:** Evaluation of variables potentially impacting progression‐free survival

	Placebo		AT‐005		
Variable	Median days	95% CI	Median days	95% CI	*p*‐Value
All dogs	103 (*n* = 25)	56–118	64 (*n* = 24)	36–118	0.8163
Stage ≤III	102.5 (*n* = 23)	56–118	69 (*n* = 16)	36–226	0.8122
Stage >III	Not estimable (*n* = 2)^a^	–	43.5 (*n* = 8)	–	–
**Histological grade**					
Intermediate grade	112 (*n* = 8)	19–118	226 (*n* = 3)	36–226	0.5182
Intermediate–high grade	102 (*n* = 5)	48–120	118 (*n* = 5)	59–upper bound not estimable	0.3560
High grade	56 (*n* = 9)	21–219	52.5 (*n* = 12)	27–upper bound not estimable	0.9702
Intermediate plus intermediate–high grade	105.5 (*n* = 13)	48–118	118 (*n* = 8)	36–upper bound not estimable	0.2570
Boxer dog (including boxer mix)	90 (*n* = 6)	35–160	118 (*n* = 8)	34–upper bound not estimable	0.2985
Non‐boxer breeds	103 (*n* = 19)	56–162	44.5 (*n* = 16)	27–84	0.2247
**US geographical area**					
East Coast	73.5 (*n* = 15)	21–117	57.5 (*n* = 12)	27–226	0.2884
Mid‐West	118 (*n* = 3)	71–upper bound not estimable	69 (*n* = 5)	21–118	0.1480
Southwest	164 (*n* = 5)	89–219	Not estimable (*n* = 4)	Not estimable	0.5408

Abbreviation: CI, confidence interval.

^a^ Both dogs were censored, thus the median progression‐free survival was not calculated.

**TABLE 4:** Evaluation of variables potentially impacting complete response percentage

Variable	Placebo (%)	AT‐005 (%)	*p*‐Value
All dogs	64	67	1.0000
Stage ≤III	65	63	1.0000
Stage >III	50	75	1.0000
**Histological grade**			
Intermediate	75	33	0.4909
Intermediate–high	80	80	1.0000
High grade	56	67	0.6731
Intermediate plus intermediate–high	77	63	0.6311
Boxer dogs (including boxer mix)	83	63	0.5804
Non‐boxer breeds	58	69	0.7267
**US geographical area**			
East Coast	53	75	0.4244
Mid‐West	100	60	0.4643
Southwest	80	75	1.000

The most common laboratory AE observed across the study population was neutropenia. Thirty‐six VCOG grade I–II neutropenic events were reported and 11 VCOG grade III–IV neutropenic events were reported. The number of neutropenic events was not different between the two treatment groups. Differences in laboratory AEs were found between the placebo and AT‐005 groups in neutrophilia and alkaline phosphatase (ALP): neutrophilia was reported in the placebo group seven times, while it was reported 15 times in the AT‐005 group. Increased ALP events were reported eight times in the placebo group and 15 times in the AT‐005 group.

The most common clinical AEs reported for both treatment groups were vomiting, weight loss, diarrhoea, lethargy, decreased appetite and anorexia. Although most of these AEs were reported to be grade I or II, for these six clinical signs, the AT‐005 group had more severe effects compared to the placebo group (29% vs. 4%).

Serious AEs were reported in three dogs during the study: one in the AT‐005 group (seizures) and two in the placebo group (febrile neutropenia and aspiration pneumonia). The evaluating investigator attributed the seizures in the dog receiving AT‐005 to progression of LSA into the central nervous system. No postmortem examinations were performed on any dog enrolled in this study. No dogs known to have the multidrug resistance gene 1 were enrolled, although testing was not required prior to enrollment.

## DISCUSSION

Previous studies evaluating L‐CHOP treatment for canine peripheral LSA, although numerous, have been retrospective in nature and included confounding variables that limited the number of evaluable T‐cell dogs treated with L‐CHOP alone.^1^, ^3^, ^4^, ^5^ The study described herein represents (to the best of the authors’ knowledge) the largest, prospective, naïve, peripheral T‐cell LSA‐only population of dogs treated with L‐CHOP chemotherapy. This generally homogenous population of T‐cell LSA dogs treated similarly will help to elucidate the true response rate and PFS of these dogs when treated with L‐CHOP, providing prospective outcomes to compare with alternative treatments in the future. The authors believe these results are even more important than the lack of impact from the mAb.

While the ORR to L‐CHOP in this population was high (98%), the PFS time for those treated with L‐CHOP and placebo was disappointingly low (103 days), findings that are similar to previous reports.^4^, ^5^ The addition of the mAb, AT‐005, did not statistically impact the PFS and, in fact, was associated with a PFS which was numerically less than the placebo group (64 days).

None of the variables evaluated (stage, histological grade, breed or geographical region) impacted the overall PFS or duration of response. Stage was dichotomised between stages II and III versus stages IV and V based on previous studies that reported a significant difference in survival time in dogs with stage III B‐cell LSA versus stage III T‐cell LSA. This study supports the finding that stage II and III T‐cell LSA has a significantly decreased survival time compared to historical stage II and III B‐cell LSA controls.^31^ For dogs where histological grade was evaluated, no difference was found between intermediate‐ or high‐grade LSA cases, as has previously been reported.^32^ Although predisposed to T‐cell lymphoma,^33^ boxer dogs were not found to have a poorer prognosis compared to all other breeds in this study. However, numbers were low and may not have reached statistical significance. Finally, canine LSA cases in the USA residing in the west have been found to have a significantly shorter PFS compared to those residing in the south and east.^34^ However, this finding was not supported in the current population of dogs.

Canine LSA is generally considered to be chemotherapy responsive. However, T‐cell LSA has consistently been shown to be refractory to typical chemotherapy treatments.^1^ Inherent or acquired resistance to CHOP^6^, ^7^, ^8^, ^9^, ^10^ has indicated that alternative treatment approaches are necessary. One potential reason for this resistance is the increased expression of ABC transporter proteins in T‐cell LSA, creating vincristine, doxorubicin and prednisone resistance.^10^ This suggests that protocols featuring alkylators, not effluxed by ABC transporter proteins, may have increased efficacy in T‐cell LSA. Unfortunately, alkylating‐heavy protocols, such as MOPP,^4^, ^14^ lomustine with prednisone^35^ and various combinations have not significantly improved outcome.^5^, ^12^ The most encouraging data were from a study that reported on a dose‐intense LOPP regimen in dogs with naïve T‐cell LSA, which resulted in a median PFS of 431 days (*n* = 35), with those achieving a CR having a numerically longer PFS (509 days).^13^ However, these dogs were clinically diagnosed with rapidly progressive T‐cell LSA and some cases could have been low grade; larger scale studies are required to confirm these initial findings.

The improved outcome in human B‐cell LSA following treatment with rituximab, and the apparent responses to alemtuzumab and brentuximab vedotin in relapsed T‐cell LSA, has ignited interest in development of a mAb for the treatment of canine LSA. AT‐005 was licensed by the USDA for dogs with T‐cell LSA based on a safety and efficacy study combining AT‐005 and a single dose of L‐asparaginase.^24^ The current expanded trial evaluating the combination of AT‐005 and L‐CHOP versus L‐CHOP alone did not result in a statistically different PFS between dogs with naïve T‐cell LSA that received the mAb and those that did not, although there was no evidence that AT‐005 negatively impacted outcome in this population. Lymphoma stage, histological grade, boxer breed and US region did not statistically impact outcome. A larger number of dogs in the AT‐005 group were classified as stage IV and V and were deemed to have a numerically higher performance score compared to the placebo group. However, numbers were small and so the impact on outcome of these variables in the AT‐005 group is difficult to define. Unfortunately, it is beyond the scope of this study to elucidate why AT‐005 was not more successful. Following initiation of this study, it was determined that AT‐005 lacks binding specificity to the intended target (CD52).^24^ Given the apparent lack of efficacy, additional studies with this particular formulation are not warranted.

The primary goal of this study was to describe the response rate of dogs diagnosed with peripheral nodal T‐cell LSA to L‐CHOP chemotherapy, with and without AT‐005. Although prospective, limitations included lack of standardised staging, leading to possible stage migration and lack of histological confirmation of high‐grade lymphoma in all cases. Nevertheless, this group of cases provides important, prospective information about adverse chemotherapy events and the outcome of dogs with peripheral nodal T‐cell LSA treated with L‐CHOP chemotherapy alone; also, it provides further evidence that the response rate and PFS of canine peripheral nodal T‐cell LSA cases to L‐CHOP chemotherapy is low. The exact mechanism for this mediocre response needs additional investigation. Given the current literature, alkylator‐heavy treatment protocols may be superior to anthracycline‐based protocols and require further, prospective evaluations in dogs with peripheral nodal T‐cell LSA.

## AUTHOR CONTRIBUTIONS

Laura S. Treml, Lydia C. Cook McAnulty and Elizabeth A. McNiel were involved in the initial study design and data collection. Laura S. Treml, Lydia C. Cook McAnulty, Elizabeth A. McNiel, Margaret L. Musser, Craig A. Clifford, Philip J. Bergman and Chad M. Johannes evaluated the data and statistical results. Margaret L. Musser initially prepared the manuscript. All authors approved the final manuscript.

## CONFLICTS OF INTEREST

Chad Johannes, Laura Treml and Lydia Cook McAnulty are former employees of Aratana Therapeutics, Inc. and were employed by Aratana Therapeutics, Inc. during the study period. Elizabeth McNiel is a former employee of Elanco Animal Health. Chad Johannes, Craig Clifford and Philip Bergman are advisory board members for Elanco Animal Health and receive honoraria. Margaret Musser has not declared any conflicts of interest.

## ETHICS STATEMENT

The authors confirm that the ethical policies of the journal, as noted on the journal's author guidelines page, have been adhered to. Global study oversight was conducted by Animal Clinical Investigation (ACI), Chevy Chase, MD, USA (www.animalci.com/). The research was approved by the ACI Animal Care and Use Committee on 25 March 2014. Institutional animal care and use documentation was also completed for those sites where it was required. Written, informed owner consent was obtained for each study dog prior to screening. This study complied with all applicable animal welfare regulations related to the humane care and use of animals.

## Data Availability

Research data are not shared.
